# PlantGSEA: a gene set enrichment analysis toolkit for plant community

**DOI:** 10.1093/nar/gkt281

**Published:** 2013-04-30

**Authors:** Xin Yi, Zhou Du, Zhen Su

**Affiliations:** ^1^State Key Laboratory of Plant Physiology and Biochemistry, College of Biological Sciences, China Agricultural University, Beijing 100193, China and ^2^Department of Bioinformatics, School of Life Sciences and Technology, Tongji University, 1239 Siping Road, Shanghai 200092, China

## Abstract

Gene Set Enrichment Analysis (GSEA) is a powerful method for interpreting biological meaning of a list of genes by computing the overlaps with various previously defined gene sets. As one of the most widely used annotations for defining gene sets, Gene Ontology (GO) system has been used in many enrichment analysis tools. EasyGO and agriGO, two GO enrichment analysis toolkits developed by our laboratory, have gained extensive usage and citations since their releases because of their effective performance and consistent maintenance. Responding to the increasing demands of more comprehensive analysis from the users, we developed a web server as an important component of our bioinformatics analysis toolkit, named PlantGSEA, which is based on GSEA method and mainly focuses on plant organisms. In PlantGSEA, 20 290 defined gene sets deriving from different resources were collected and used for GSEA analysis. The PlantGSEA currently supports gene locus IDs and Affymatrix microarray probe set IDs from four plant model species (*Arabidopsis thaliana*, *Oryza sativa*, *Zea mays* and *Gossypium raimondii*). The PlantGSEA is an efficient and user-friendly web server, and now it is publicly accessible at http://structuralbiology.cau.edu.cn/PlantGSEA.

## INTRODUCTION

Recent revolution in sequencing technology leads to ever-increasing high-throughput data, and the tremendous repository of publicly available data becomes an invaluable treasure. Meanwhile, the novelty and complexity of exploiting these data highlighted the challenges in computational analysis and knowledge mining. One of the popular methods is Gene Set Enrichment Analysis (GSEA) (1), which is a promising method to interpret biological meaning by calculating the overlaps between the requested gene lists and various previously defined gene sets, and the gene lists can be derived from high-throughput experiments like microarray or next-generation sequencing. Gene Ontology (GO) system (2) is a popular annotation system for defining gene set on which many tools were developed toward different groups of users in the scientific community. EasyGO (3) and agriGO (4), two GO enrichment analysis tools developed by our laboratory, focus on agricultural organisms and have accomplished >25 000 and >60 000 analysis requests from users around the world, respectively. During the process of maintaining these tools, we realized that the coverage of GO annotated genes is limited because of their prediction method and systematic structure, whereas GSEA analysis turns out to be a method that can broaden the scope of data set and make up the deficiency of GO. Thus, to meet the increasing requirements of more comprehensive analysis, we developed a newly designed web server named PlantGSEA implementing GSEA analysis for plant species.

So far, we collected 20 290 gene sets deriving from four different types of resources, including (i) well-recognized annotation systems, such as GO (2) and Kyoto Encyclopedia of Genes and Genomes (KEGG) (5), (ii) public databases, such as TAIR (6) and RGAP (7), (iii) published literatures, and (iv) computational predictions using existed tools, for example, miRNA target prediction results. Notably, we manually collected many gene sets from literatures and considered them as valuable treasure for the enrichment analysis in terms of their high accuracy and reliability.

The PlantGSEA analysis supports both gene locus IDs and Affymatrix microarray probe set IDs as input query, and it gives users a comprehensive analysis report, which can be explored and downloaded freely. Some new features have been added to extend the functionality of the PlantGSEA. For example, we provided a hierarchy visualization tool by which significantly enriched gene sets can be selected for a hierarchy analysis (Supplementary Figure S1). Based on that, users can decrease the number of total gene sets and then pay more focus on interesting ones and achieve a better understanding of relationships among different biological processes. A treeview-like navigation interface is also provided for gene sets exploration. Users can browse all the gene sets grouped by their sources and functions in one species. Collected gene sets in PlantGSEA are available for downloading in tab-separated text to facilitate relevant researchers. Last but not the least, a conversion tool, which can convert among gene locus, gene symbol and Affymatrix probe set, was developed to extend the diversity of input type in PlantGSEA.

## WEB SERVER CONSTRUCTION

### Gene sets collection and preparation

To provide a comprehensive analysis, we collected >20 000 gene sets of four plant model organisms including *Arabidopsis*, rice, maize and cotton from various resources. We grouped the gene sets into four categories, GO-related gene sets, gene family-based gene sets, curated gene sets and motif information-based gene sets on the basis of their characteristics. A statistical summary of collected gene sets in each category is presented in Table 1.

**Table 1.** Total numbers and sources of categorized gene sets in four comprehensively annotated species

Organism	Category	Number of gene sets	Source
*Arabidopsis thaliana*	GO gene sets	7041	TAIR (6)
	Gene family-based gene sets	1018	TAIR/PlantUPS (13)
	Curated gene sets	1806	AraPath (10)/literature
	Motif gene sets	764	AraPath
*Oryza sativa*	GO gene sets	2782	AgriGO (4)
	Gene family-based gene sets	118	RGAP (7)/PlantUPS
	Curated gene sets	740	Gramene (8)/KEGG (5)/POC (14)/literature
	Motif gene sets	2566	Prediction from PMRD(12)
*Zea mays*	GO gene sets	1790	AgriGO
	Gene family-based gene sets	81	PlnTFDB (11)/PlantUPS
	Curated gene sets	410	Gramene/literature
*Gossypium raimondii*	GO gene sets	6764	Phytozome (9)
	Curated gene sets	2315	Phytozome/literature

First, GO-based gene sets are most widely used in the GSEA, and we downloaded them from related annotation databases, including TAIR (6), MSU rice (7) and Phytozome (9). The GO gene sets are re-computed to cover every GO term and are prepared as gene set annotation files. Plant Ontology (PO), a controlled vocabulary describing plants’ anatomy and morphology and development, was collected from Plant Ontology Consortium (14).

A gene family means a set of genes formed by duplication of a single original gene, and generally, genes in one gene family share similar biochemical function. We collected all gene families of *Arabidopsis* and rice from TAIR and MSU, respectively, and included them as gene family-based gene sets category. Additionally, we collected transcription factor families from PlnTFDB (11) and the ubiquitin proteasome system-related gene families from plantUPS (13).

Third, we included gene sets derived from KEGG database (5), PlantCyc (8) and literatures as curated gene sets. We manually collected gene sets from published references and the AraPath database (10). In aim to clarify the gene sets, we named these gene sets based on the description in the references. For example, a set of genes co-expressed with OsWRKY22 that plays a role in the resistance response to blast was named as ‘ABBRUSCATO_CO-EXPRESSED_WITH_OSWRKY22_RESISTANCE_TO_BLAST’ beginning with the author’s name, and similarly, ‘COUDERT_CRL1_CROWN_ROOT_DEVELOPMENT_UP’ means a list of upregulated genes in crown rootless1 mutant (CRL1) associated with crown root development (15,16). Additional information, including a brief description of the gene set, a PubMed ID, abstract, authors and their affiliation and a link of publication, was also gathered and accessible in PlantGSEA. Till now, >1100 gene sets from 245 references were collected and used for the GSEA analysis, and we are committed to update our gene sets regularly in the future.

Finally, for the motif information-based gene sets, we included transcription factor targets and microRNA targets of *Arabidopsis* obtained from AraPath database and predicted microRNA targets of rice using the same approach as in our plant microRNA database (PMRD) (12).

By adopting annotations from diverse resources, we found that the proportion of annotated genes increased in a promising way comparing with of single GO annotation (Supplementary Table S1). In *Arabidopsis* and rice, the coverage of all genes increased from 79 to 91% and 32 to 63%, respectively. In cotton and maize, the proportion increased from 65 to 72% and 39 to 48%, respectively. Overall, the substantial increase of annotation coverage proved the advantage of the GSEA concept that guarantee a better understanding of biological meaning in the enrichment analysis.

### Data processing

The PlantGSEA is an easy-to-use web server that users are only required to submit a list of genes for the enrichment analysis. Users can choose different background as reference, either a pre-calculated background covering whole genome or a customized background provided by users. Both gene locus ID and probe set ID are supported by PlantGSEA. The PlantGSEA provided three statistical tests for the analysis including hypergeometric test, Fisher’s exact test and *χ*^2^ test. A statistical formula of default method, Fisher’s exact test, is displayed as follow.

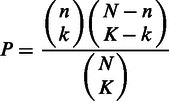



In the formula, *N* is the total number of one organism’s all genes or of the user-provided background, *n* is the number of genes in the query list, *K* means the total number of genes in one gene set and *k* stands for the number of overlapped genes. Multiple test correction is performed because of the large number of our collected gene sets and to alleviate possible false-positive rate. We provide six multiple test correction methods in PlantGSEA, including Yekutieli, Bonferroni, Hochberg, Hochberg, Hommel and Holm. We get all the adjusted *P*-values after performing false discovery rate correction and display the most significantly enriched gene sets using a cut-off selection.

### Implementation

The PlantGSEA is constructed on a Dell Server with Red Hat Enterprise Release 5.2 operating system. Analysis codes are compiled with Python (www.python.org), and the web interface is supported by PHP scripts (www.php.net). We used R software (www.r-project.org) for statistical analysis and the dot program of Graphviz software (www.graphviz.org) for generating hierarchy charts. No software or plug-in is needed to install, as the tool is web-based. The users are free to access the analysis results with a given job session after the submission of the job, and we will keep the user’s records for 3 months for their retrieving.

## SAMPLE TEST AND RESULTS ANALYSIS

We selected 174 *Arabidopsis* gene locus, which showed significantly upregulated expression pattern after cold treatment, to demonstrate the reliability of analysis results. We used the default parameters (Fisher exact Test and Yekutieli adjusted method) in GSEA analysis.

**Figure 1.**
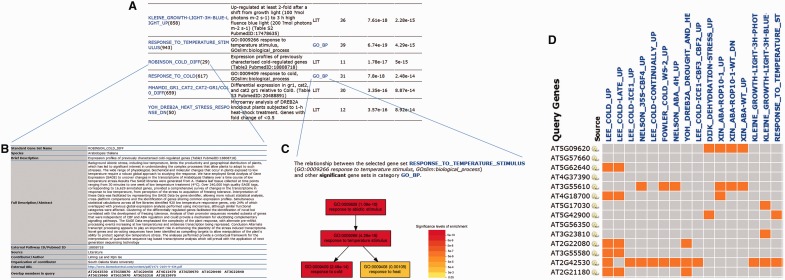
The result page in PlantGSEA. (**A**) A summary table displayed enriched gene sets with six columns: gene set names, a brief description, the category of the gene set, the number of overlap between queried genes and the gene set, *P*-value of statistical test and adjusted *P*-value after the False Discovery Rate (FDR) correction. (**B**) Detail information about the selected gene set, including gene set name, brief and full description, contributor’s information and overlapped genes. (**C**) The enriched gene sets displayed in the hierarchical pattern. The color showed the significance of a gene set. (**D**) An annotation table represented the gene set-based annotation of query genes. Gene sets and queried genes can be further explored for detail information by clicking the links.

Two main components are shown in the result page: a summary table of statistically enriched gene sets (Figure 1A) and a gene annotation table displaying the overlaps between user-submitted genes and our pre-defined gene sets (Figure 1D). In the summary table, we provided the detailed description (Figure 1B) and hierarchy analysis (Figure 1C) for each enriched gene set. In the gene annotation table, users can explore the query genes that overlap with different gene sets, and outside links of the genes for more detailed information are available. The table is designed to facilitate the users for having an overall and comprehensive understanding of their gene lists. Additionally, we provide users a basic summary, which includes the query ID redundancy report, non-redundant query list and categories user chose to analyze.

**Figure 2.**
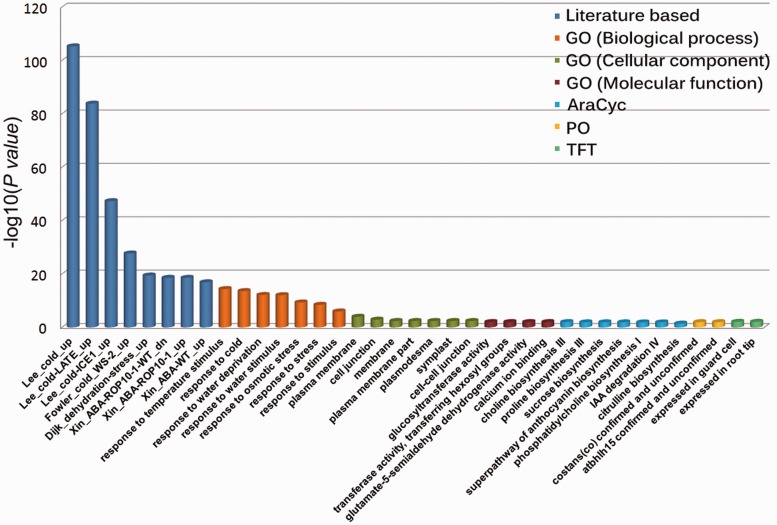
A Bar chart of significantly enriched gene sets in different categories. These terms were selected from significantly enriched gene sets (FDR < 0.05). The *y*-axis represented the value of log10-converted adjusted *P*-value. The *x*-axis displayed the gene sets name. Gene sets belonging to different categories were marked with different colors. GO, gene ontology; PO, plant ontology; TFT, transcription factor target; FDR, False Discovery Rate.

The significance of enriched gene sets selected from each category has been showed in Figure 2. As displayed, the sample showed to be significantly enriched in gene sets associated with cold and stimulus response processes, for instance, the GO-based gene set ‘response to cold’ and ‘response to temperature stimulus’, the AraCyc-based gene sets ‘proline biosynthesis III’ and TFT-based gene sets ‘atbhlh15 confirmed and unconfirmed’. It is worth to note that the query gene displayed exceedingly significant overlaps with the literature-based gene sets like ‘Lee_cold_up’, ‘Lee_cold-ICE1_up’ and ‘Xin_ABA-ROP10-1-WT_dn’, suggesting that the expression of this gene list may not only be related to cold response but also regulated by ICE1 [inducer of C-repeat binding factor (CBF) expression 1] or involved in ABA signaling regulated by ROP10 GTPases (AT3G48040.1). The result demonstrated the high accuracy and sensitivity of our literature-based GSEA method.

## DATA ACCESSION AND OTHER FUNCTIONS

The collected gene sets in PlantGSEA are downloadable, making it a valuable annotation resource for the community. We further developed a tool named Format Conversion Tool for converting IDs among gene locus, gene symbol and Affymatrix probe set (Supplementary Figure S3). Users can submit either a list of locus IDs or probe set IDs and convert them to IDs of different format. Furthermore, we provided a convenient way for users to browse all gene sets in a species. We grouped gene sets into four categories as described earlier in the text, and developed a treeview-like navigation interface (Supplementary Figure S2), based on which users can check detailed information of gene sets.

## DISCUSSION

We provided a GSEA-based tool named PlantGSEA to facilitate the analysis and interpretation of high-throughput data for the plant species. Besides newly designed back-end structure and comprehensive exploration and visualization interface, PlantGSEA was featured by collecting and applying >20 000 gene sets from diverse resources in the analysis. According to Huang *et al.* (17), the GSEA method we used had been approved as of many advantages comparing with singular enrichment analysis (SEA)-based method like GO enrichment analysis because GSEA had a more extensive annotation foundation and, accordingly, gained a better chance to uncover biological facts. As demonstrated in the Figure 2, indeed, the reference-based gene sets could generate a more convincible and comprehensive analysis result than SEA-based method (Supplementary Figure S4) (4).

The gene sets collection is still an ongoing work as new annotations and references appear continuously. The PlantGSEA currently supports four species. Same as our previous EasyGO and agriGO tools, the PlantGSEA will be under active updating and regular maintenance. New species or new genome annotations will be included in every new release of PlantGSEA or upon users’ request. Further work that grouping gene sets with similar meaning into the cluster may also be valuable for the PlantGSEA platform.

How to properly interpret the high-throughput data has been a question involving in different aspects of thinking and different analytical methods. Our series of analysis toolkit, EasyGO, agriGO and PlantGSEA contribute to uncovering facts from the data by calculating the enrichment gene ontology or gene sets within the query gene lists. The future work will lie in the integrative analysis of different types of data, for example, chromatin immunoprecipitation followed by high-throughput sequencing (ChIP-seq) data, DNaseI hypersensitive sites sequencing (DNase-seq) data and mutation information and so forth. It is conceivable that our toolkit series together with other analytical tools may shed light on revealing the multifaceted relationship and the regulatory mechanisms of genes in plants.

In summary, the PlantGSEA is a comprehensive and easy-to-use GSEA-based web server in aim to serve plant research community, and we believe a large amount of researchers will benefit from it.

## SUPPLEMENTARY DATA

Supplementary Data are available at NAR Online: Supplementary Table 1 and Supplementary Figures 1–4.
